# Role of Tristetraprolin in the Resolution of Inflammation

**DOI:** 10.3390/biology10010066

**Published:** 2021-01-19

**Authors:** Peter Rappl, Bernhard Brüne, Tobias Schmid

**Affiliations:** 1 Institute of Biochemistry I, Faculty of Medicine, Goethe-University Frankfurt, 60590 Frankfurt, Germany; rappl@biochem.uni-frankfurt.de (P.R.); B.Bruene@biochem.uni-frankfurt.de (B.B.); 2 German Cancer Consortium (DKTK), Partner Site Frankfurt, 60590 Frankfurt, Germany; 3 Frankfurt Cancer Institute, Goethe-University Frankfurt, 60596 Frankfurt, Germany; 4 Project Group Translational Medicine and Pharmacology TMP, Fraunhofer Institute for Molecular and Applied Ecology, 60596 Frankfurt, Germany

**Keywords:** RNA-binding protein, AU-rich element, post-transcriptional regulation, mRNA stability

## Abstract

**Simple Summary:**

Chronic inflammatory diseases account for up to 60% of deaths worldwide and, thus, are considered a great threat for human health by the World Health Organization. Nevertheless, acute inflammatory reactions are an integral part of the host defense against invading pathogens or injuries. To avoid excessive damage due to the persistence of a highly reactive environment, inflammations need to resolve in a coordinate and timely manner, ensuring for the immunological normalization of the affected tissues. Since post-transcriptional regulatory mechanisms are essential for effective resolution, the present review discusses the key role of the RNA-binding and post-transcriptional regulatory protein tristetraprolin in establishing resolution of inflammation.

**Abstract:**

Inflammation is a crucial part of immune responses towards invading pathogens or tissue damage. While inflammatory reactions are aimed at removing the triggering stimulus, it is important that these processes are terminated in a coordinate manner to prevent excessive tissue damage due to the highly reactive inflammatory environment. Initiation of inflammatory responses was proposed to be regulated predominantly at a transcriptional level, whereas post-transcriptional modes of regulation appear to be crucial for resolution of inflammation. The RNA-binding protein tristetraprolin (TTP) interacts with AU-rich elements in the 3′ untranslated region of mRNAs, recruits deadenylase complexes and thereby facilitates degradation of its targets. As TTP regulates the mRNA stability of numerous inflammatory mediators, it was put forward as a crucial post-transcriptional regulator of inflammation. Here, we summarize the current understanding of the function of TTP with a specific focus on its role in adding to resolution of inflammation.

## 1. Introduction

Effective immune responses towards pathogens or injury are characterized by acute inflammations aimed at eliminating the inciting stimulus. To prevent inefficient removal of the threat but also excessive damage to the host, inflammatory processes must be tightly controlled. Thus, in addition to transcriptional regulation contributing to de novo expression of inflammatory mediators, a strict post-transcriptional regulatory program ensures the timely and quick adaptation of the responses to rapidly changing environmental cues in the progress of inflammation [1,2].

## 2. Inflammation

### 2.1. The Course of Inflammation

**Figure 1:**
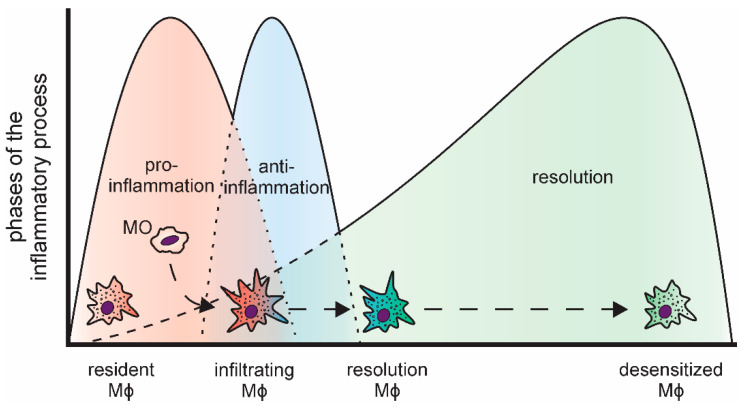
Distinct phases of an acute, transient inflammatory process and the respective macrophage subtypes (MO: monocyte; Mϕ: macrophage).

Inflammatory reactions are important to combat invading pathogens [3,4]. Nevertheless, to prevent local tissue damage due to the sustained production of highly reactive inflammatory mediators, including reactive oxygen species and nitric oxide [5], and to reduce the risk of systemic effects, inflammatory responses need to be resolved in a timely manner [6]. In line, the course of acute inflammations is tightly regulated and follows a common pattern (Figure 1). Bacterial or viral pathogens, i.e., pathogen-associated patterns (PAMPs), are commonly detected by tissue-resident macrophages, which constitute the first line of defense, via so-called pattern-recognition receptors (PRRs) [7,8]. Similarly, tissue damage elicits an inflammatory response in local phagocytes by activating PRRs via damage-associated molecular patterns (DAMPs), such as intracellular components released upon necrotic cell death [9,10]. The most prominent family of PRRs are the Toll-like receptors (TLRs). They activate intracellular signaling cascades including kinases (e.g., mitogen-activated protein kinases (MAPKs)) and transcription factors (e.g., nuclear factor κ B (NFκB)) to coordinate the inflammatory responses [11,12]. Upon activation, resident macrophages rapidly recruit neutrophils, which spear-head the pro-inflammatory responses [13,14,15]. Neutrophils are closely followed by infiltrating monocytes, which subsequently differentiate into macrophages within the respective tissue [16]. In line with their versatile regulatory functions, macrophages are a highly plastic cell type [17]. Upon activation, they present an inflammatory, classically activated phenotype, characterized by production of pro-inflammatory cytokines, such tumor necrosis factor (TNF), interleukin 6 (IL6), or IL8 [18,19]. During the progression of the inflammatory process and triggered by the phagocytosis of the rapidly dying neutrophils, they take on an alternatively activated phenotype producing anti-inflammatory mediators, e.g., IL10 [20,21,22,23]. The last phase of an acute, transient inflammation is aimed at resolving the inflammatory environment, both pro- and anti-inflammatory components, and reestablishing cellular and humoral homeostasis at the site of inflammation [24,25]. Importantly, while resolution was considered a rather passive event for a long time, it is now widely accepted to be a highly regulated, complex process, which is initiated already early on during the course of inflammation [26,27]. In fact, successful resolution is not the end of this dynamic process, but recent reports indicate that it is followed by a prolonged post-resolution phase characterized by an altered, immune suppressive environment [28,29].

As indicated above, macrophages fulfill a central role in the execution of inflammatory responses. Interestingly though, macrophage populations change massively during inflammation, i.e., the original tissue resident macrophage population of mesodermal origin is rapidly reduced upon influx of neutrophils, both by cell death and emigration [30], and macrophages differentiating from infiltrating monocytes emerge to replenish the resident macrophage pool [31,32].

### 2.2. Post-Transcriptional Regulation in Inflammation

While typical transcriptional changes characterize inflammatory responses brought about, e.g., by transcription factors of the STAT and NFκB families [33,34], expression of many inflammatory mediators is subject to tight post-transcriptional control as well. The latter coordinates the emergence and, even more importantly, the timely termination of the production of certain factors [33,35]. The specificity of such mechanisms is achieved by intricate regulatory principles encoded within the sequence of individual mRNAs and by a wealth of *trans*-acting factors such as non-coding RNA species [36] and RNA-binding proteins (RBPs) [37,38]. For example, roquin-1 and regnase-1 (monocyte chemotactic protein-induced protein 1 (MCPIP1)) have been shown to be induced in macrophages by inflammatory stimuli and in turn regulate inflammatory responses in a coordinate manner [39,40,41]. Specifically, regnase-1 is an early response gene, and via its endonuclease function, cleaves actively translated inflammatory target mRNAs during the early phase of inflammation to limit the extent of the inflammatory reactions [42,43,44]. In line, mice deficient for regnase-1 develop spontaneous inflammatory syndromes, likely due to uncontrolled immune responses to external pathogens as these could be these could be attenuated by antibiotics treatment [45]. In contrast, roquin-1 was shown to degrade translationally inactive mRNAs stored in stress granules or processing bodies, thereby tuning inflammatory gene expression during later phases to prevent chronification of inflammatory process [46,47]. Interestingly, failure to localize to processing bodies due to mutational inactivation of the respective domain provoked a hyperinflammatory phenotype in mice and was even described for a human case [48]. Of note, recent studies further identified a huge number of unconventional RBPs, many of which were primarily characterized as metabolic enzymes (e.g., glyceraldehyde-3-phosphate dehydrogenase) carrying-out additional “moonlighting” functions [49,50], stressing the tight interplay between metabolism and post-transcriptional regulation. Considering the elevated energetic demand in inflammatory settings [51,52], it can be speculated that some of the not yet characterized RBPs might be involved in the post-transcriptional control of inflammatory mediators as well.

Amongst the classical RBPs, tristetraprolin (TTP) emerged as one of the most prominent post-transcriptional regulators of immune functions. Here, we want to provide a brief update of the role of TTP during the course of inflammation, specifically focusing on its contribution to an effective resolution phase.

## 3. Tristetraprolin

### 3.1. TTP Structure and Function

**Figure 2:**
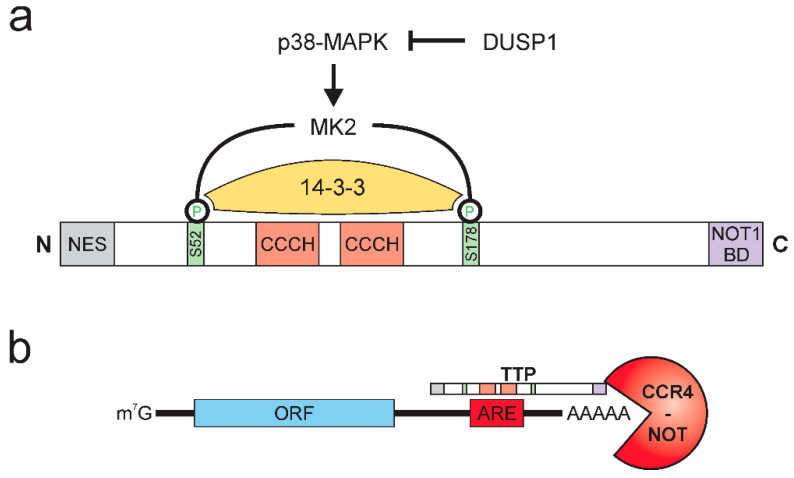
Tristetraprolin (TTP) protein structure, activity regulation, and function. (**a**) p38-MAPK activates MK2, which phosphorylates TTP at serines 52 and 178 (mouse), which allows for binding of 14-3-3 and inactivation of TTP. DUSP 1 inhibits p38-MAPK. (NES: nuclear export signal; NOT1 BD: NOT1 binding domain) (**b**) TTP interacts with adenosine and uridine (AU)-rich elements (AREs) in the 3′ untranslated region of target mRNAs and recruits the CCR4-NOT deadenylation complex, facilitating rapid degradation of the target mRNA. (ORF: open reading frame).

TTP (or ZFP36) was first described in 1991 [53] and belongs to a highly conserved family of zinc-finger proteins with RNA-binding properties [54]. In addition to domains facilitating its nuclear-cytoplasmic shuttling, TTP contains a C-terminal NOT1 binding domain and, alike other members of the TTP family, two tandem CCCH-type zinc-finger domain structures. The activity of TTP is pre-dominantly regulated by post-translational modifications, affecting stability, localization, and DNA binding properties of TTP [55,56,57,58]. The phosphorylation of TTP by p38-MAPK-activated protein kinase 2 (MK2) emerged as the major regulator of TTP activity [59,60]. Specifically, MK2 phosphorylates TTP at two serine residues (mouse: S52 and S178; human: S60 and S186), thereby creating binding sites for the chaperone 14-3-3, which prevents localization of TTP to stress granules, thereby inhibiting the mRNA destabilizing activity of TTP [61,62]. Activation of TTP can be achieved by various phosphatases [63], while dual-specificity phosphatase 1 (DUSP1) was shown to prevent TTP inactivation by inhibiting p38-MAPK [64,65], protein phosphatase 2A (PP2A) dephosphorylates and thereby prevents interaction with 14-3-3 [66,67]. As a side note, *Dusp1* itself is a target of TTP-mediated destabilization. Consequently, inactivation of TTP by p38-MAPK at the same time enhances expression of the p38-MAPK inhibitor DUSP1, providing a negative feedback mechanism to ensure tight and rapid regulation of inactivation and reactivation of TTP [68] (Figure 2a).

The CCCH domains constitute the RNA-interaction interphase of TTP [41]. They allow TTP to specifically interact with adenosine and uridine (AU)-rich elements (AREs) [69], which are commonly found within 3′ untranslated regions (3′ UTRs) of mRNAs and have been described as important RNA stability regulatory platforms for a broad group of ARE-binding proteins [70,71]. While the ARE-binding properties of TTP have been described early after its discovery [72,73], recently, global interaction mapping studies suggested preferential binding of TTP to nonameric UUAUUUAUU motifs or heptameric UAUUUAU motifs [74,75,76]. Upon binding to its target mRNAs, TTP recruits the CCR4-NOT deadenylation complex via its C-terminal NOT1-binding domain [77,78,79,80], thereby allowing for deadenylation and subsequent 3′-5′ exosomal degradation of the respective target mRNA [81] (Figure 2b).

In addition to the mRNA destabilizing properties, TTP was also shown to inhibit the translation of mRNAs [82,83,84]. The finding that TTP is able to regulate its targets at various post-transcriptional levels underscores the notion that such regulatory and redundant principles are required to rapidly and efficiently regulate the timely expression of specific targets [75,85].

### 3.2. TTP in the Resolution of Inflammation

TTP activity is tightly regulated during the course of inflammation via the inhibitory activity of p38-MAPK [59], but also by autoregulation [86]. To assess how TTP contributes to the regulation of inflammation, various groups have used RNomics approaches to identify mRNAs both directly bound and regulated by TTP in bone marrow-derived macrophages (BMDM) in response to inflammatory stimulation with LPS [37,74,75]. In the following, we will discuss the combined findings to provide an overview of TTP targets from the onset (1 h LPS) via the peak (3 h LPS) to the early resolution of inflammation (6 h LPS).

Since TTP is inactivated early during inflammatory simulation, Tiedje et al. determined TTP targets under these conditions using an individual-nucleotide resolution crosslinking and immunoprecipitation (iCLIP) approach using TTP-knockout (TTP-k/o) BMDMs overexpressing either wildtype TTP (TTP-wt) or a phosphorylation-site (S52/178A) TTP mutant (TTP-AA), which is inactivation-resistant [37]. Using this approach, they identified 4106 mRNAs to be bound by TTP within their 3′UTRs. Interestingly, only for a small proportion of these TTP-interacting mRNAs enhanced LPS-induced expression was sensitive to expression of the constitutively active TTP-AA. Specifically, 8 mRNAs showed higher expression in TTP-wt as compared to TTP-AA expressing BMDMs after 1 h LPS treatment (Table 1; *left columns*). Amongst these, immediate early response 3 (*Ier3*), a feedback inhibitor of NFκB signaling [87], appeared to benefit most from TTP inactivation in TTP-wt cells. In line, the authors identify altered NFκB activation signatures in TTP-AA expressing macrophages. Similarly, urokinase-type plasminogen activator (*Plau*) mRNA appeared to be stabilized when TTP was inactivated in TTP-wt cells. PLAU was previously shown to act anti-inflammatory by suppressing fibrin-deposition induced inflammation [88] and inflammatory osteoclastogenesis [89]. These findings suggest that inactivation of TTP, rather than providing an additional boost to inflammatory targets, post-transcriptionally derepresses feedback inhibitors of inflammation and anti-inflammatory targets, thereby setting the stage for successful resolution already very early on during the inflammatory process (Figure 1). In line, high levels of active TTP at this stage of inflammation have been shown to enhance the stability of certain ARE-containing transcripts, such as *Tnf* and *Cxcl2*, supporting the concept that a proper resolution phase requires an intact inflammatory phase, also at the level of TTP activity regulation [90].

While TTP is inactivated during the onset of inflammation, it becomes gradually reactivated during the progression of inflammation by autoregulatory feedback loops. Specifically, as TTP destabilizes its own mRNA, inactivation of TTP allows for enhanced expression of TTP [86]. At the same time, the TTP target DUSP1 increases, which inhibits p38-MAPK, thereby allowing for activation of TTP [68]. Thus, the TTP-interacting targets determined by Sedlyarov and colleagues using a photoactivatable- (PAR-) iCLIP approach for TTP in BMDMs after 3 and 6 h of LPS served as indicators for later stages of the inflammatory course [74]. In line with the predicted TTP activation pattern, the number of TTP targets responsive to changes in the availability of TTP (i.e., TTP-wt vs. TTP-k/o) increased. In addition to *Ier3*, which appeared to be a highly regulated TTP target throughout the course of inflammation, *Cxcl1* and *Cxcl2* showed a strong increase in expression in TTP-deficient (TTP-k/o) BMDM at peak inflammation (3 h LPS; Table 1, *middle columns*). *Cxcl1* and *Cxcl2* are well-established TTP targets [91,92], both being important chemokines contributing to early stage neutrophil recruitment [93]. The observation that the reduction in neutrophil-attracting chemokines in the presence of active TTP in TTP-wt cells even precedes the impact of TTP on early response pro-inflammatory cytokines, such as *Tnf*, provides further evidence for a tightly regulated, TTP-controlled resolution program, which is initiated already during the onset of inflammation [94]. The well-known anti-inflammatory TTP target *Il10* [95,96,97] appeared to be downregulated by TTP also already during the presumed pro-inflammatory phase.

Moving on to early resolution of inflammation (6 h LPS), not only the number of mRNAs bound and regulated by TTP but also the degree of TTP-dependent regulation massively increased (Table 1, *right columns*). In addition to the neutrophil-targeting chemokines *Cxcl1* and *Cxcl2*, further chemokines including *Ccl2* [98], *Ccl3* [99], and *Ccl4* [100] were down-regulated by TTP at the start of the resolution phase. While CCL3 attracts and activates neutrophils and also monocytes and macrophages, CCL2 rather recruits monocytes, memory T cells, and dendritic cells, while CCL4 primarily attracts natural killer cells and monocytes [101,102]. Thus, TTP appears to initiate the normalization of the cellular environment at the site of inflammation very early during the resolution. Of note, while *Tnf* was established as a TTP target early on [72,98,103,104,105], it only emerged as TTP-bound and -responsive target after 6 h of LPS in the present studies, while it was not regulated by TTP at 1 or 3 h of LPS. This is in line with previous reports showing that *Tnf* accumulates maximally in BMDM at 3 h of LPS declining only thereafter [1,75].

Corroborating the importance of TTP for the onset of resolution, Joe et al. recently showed that CD38 induces TTP expression already during early inflammation via its products nicotinic acid adenine dinucleotide phosphate (NAADP) and cyclic ADP ribose (cADPR). As part of a feedback loop, increasing TTP levels destabilize *Cd38*, which results in the accumulation of nicotinamide adenine dinucleotide (NAD^+^), the pre-cursor of the abovementioned second messengers. Elevated NAD^+^ concentrations then activate SIRT1, which enhances TTP activity by deacetylation during the onset of resolution. In addition to the regulation of cytokines, TTP inhibits the expression of the mTOR-activator RHEB, thereby inducing autophagolysosome formation, which is crucial for bacterial clearance [106]. In contrast, deletion of TTP was recently shown to protect neutrophils from apoptosis, which enhanced their anti-microbial activity [107].

**Figure 3:**
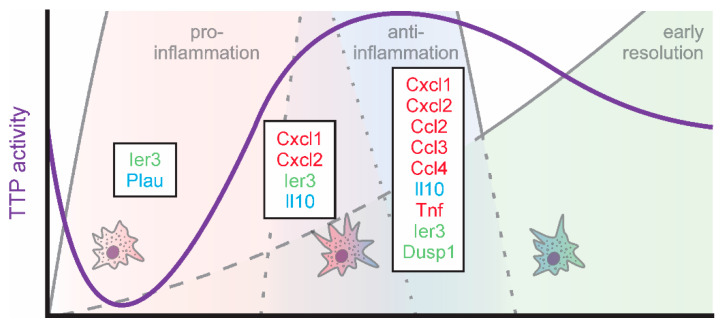
TTP activity (violet) and targets (green: feedback regulators; red: inflammatory; blue: anti-inflammatory) in macrophages during the early phases of inflammation.

Taken together, TTP emerges as a regulator of resolution, by coordinating the regulation of the early phases of resolution, which are initiated already immediately after inflammatory stimulation (Figure 3).

Nevertheless, while in vitro studies provide important mechanistic and regulatory insights regarding the function of TTP, various mouse models have been generated to study the impact of TTP in vivo. In line with the inflammation-regulatory function of TTP, mice with a global TTP-knockout develop a severe inflammatory disease phenotype soon after birth [108,109]. In contrast, mice with a myeloid-specific depletion of TTP exhibit only a minimal spontaneous phenotype but appear hypersensitive against inflammatory challenge [110]. Furthermore, mice expressing only the non-phosphorylatable, constitutively active TTP-AA mutant showed a strongly attenuated inflammatory response to LPS [111]. Nevertheless, these mice were also not able to develop a desired inflammatory immune response against infections with *Salmonella typhimurium* [112], indicating that tunable TTP is crucial for the regulation of appropriate immune responses. Interestingly, deletion of the AREs within the TTP 3′UTR sufficed to increase the expression of TTP and protected respective mice against a number of murine models of human diseases, including rheumatoid arthritis, psoriasis, and multiple sclerosis [113].

## 4. Conclusions and Future Directions

TTP has been extensively studied with respect to its role in inflammation and has long been assumed to be an exclusively anti-inflammatory factor, based both on the severe inflammatory phenotype of the respective knockout mice and its wealth of inflammatory targets. Nevertheless, in the meantime, it has been acknowledged that its function is not quite that unambiguous as TTP also attenuates the expression of anti-inflammatory mediators. Considering its tight regulation throughout the course of inflammation and its broad spectrum of targets, TTP might rather be assigned as a master regulator of the resolution of inflammation ensuring a well-coordinated and timely return to homeostasis after an inflammatory insult.

Consequently, modulating TTP might provide a promising avenue for the development of inflammation regulatory therapies. Specifically, therapeutic approaches to increase the TTP activity can be expected to ameliorate the course of numerous chronic inflammatory diseases with a previously shown connection to TTP, such as rheumatoid arthritis [108,114,115], atherosclerosis [116], diabetes [117], inflammatory bowel disease [118], chronic obstructive pulmonary disease [119], and autoimmune diseases [108,120]. Since chronic inflammatory diseases are a major health care burden, such approaches appear extremely appealing. For example, PP2A agonists were shown to activate TTP and consequently reduced inflammation and bone loss in a murine rheumatoid arthritis model [121]. Further, small molecules could be screened for their potential to enhance TTP activity either by increasing its transcription or its mRNA stability or by preventing its inactivation. Nevertheless, such approaches commonly are limited by off-target effects. Considering the self-regulatory properties of TTP, the design of steric-blocking oligonucleotides to prevent the interaction of TTP with its own mRNA should enhance TTP expression [122]. In contrast, TTP-inhibitory approaches should intensify and prolong inflammatory processes and therefore are predicted to favor acute anti-bacterial reactions [107]. Nevertheless, considering the important function of TTP in the resolution of inflammation, TTP-inhibitory anti-infectious therapies should be developed with caution. Irrespective of the direction, potential therapeutic endeavors will have to factor in the cell type-specificity of TTP targets and the so far largely neglected interplay between TTP and other *trans*-acting regulators including RBPs and miRNAs.

It will further be of great interest to see if polymorphisms in TTP exist in humans, which might serve as markers for the individual susceptibility to certain chronic inflammatory diseases.

## Figures and Tables

**Table 1:** Differential expression of TTP-bound mRNAs during different phases of inflammation.

Inflammatory Onset ^1^		Peak Inflammation ^2^		Early Resolution ^3^	
Gene ID	log2FC (TTP-wt vs. TTP-AA)	Gene ID	Δ log2FC (TTP-ko/ctr vs. TTP-wt/ctr)	Gene ID	Δ log2FC (TTP-ko/ctr vs. TTP-wt/ctr)
*Ier3*	0.91	*Gdf15*	1.73	*Cxcl1*	3.86
*Plau*	0.57	*Eno2*	1.26	*Cxcl2*	3.34
*Lpl*	0.56	*Mllt11*	1.23	*Eno2*	2.07
*Mdm2*	0.46	*Cxcl1*	1.00	*Mllt11*	1.73
*Mat2a*	0.44	*Cxcl2*	0.94	*Ccl3*	1.46
*Plek*	0.36	*Rusc2*	0.67	*Il10*	1.39
*Canx*	0.35	*Ier3*	0.45	*Tnf*	1.36
*Actr2*	0.26	*Zeb2*	0.26	*Rusc2*	0.87
		*Il10*	0.25	*Il27*	0.74
		*Prdx1*	0.20	*Notch1*	0.50
		*Notch1*	0.14	*Maf*	0.35
		*Sfmbt1*	0.13	*Ccl4*	0.35
		*Cish*	0.03	*Fam49b*	0.33
		*Cflar*	0.02	*Cdkn1a*	0.28
				*Zeb2*	0.28
				*Ier3*	0.23
				*Il10ra*	0.21
				*Dusp1*	0.05
				*Ccl2*	0.05
				*Aff1*	0.02

Green: feedback regulators; red: inflammatory TTP targets; blue: anti-inflammatory TTP targets. ^1^ Top 50 3′UTR-bound TTP targets, filtered for targets with higher induction of mRNA expression in TTP-wt as compared to inactive TTP-AA expressing BMDM after 1 h LPS (from [37]). ^2^ Top 50 3′UTR-bound TTP targets, filtered for targets with higher induction of mRNA expression in TTP-k/o as compared to TTP-wt BMDM after 3 h LPS (from [74]). ^3^ Top 50 3′UTR-bound TTP targets, filtered for targets with higher induction of mRNA expression in TTP-k/o as compared to TTP-wt BMDM after 6 h LPS (from [74]).
